# Editorial: Nurturing medical professionalism in different cultural contexts

**DOI:** 10.3389/fmed.2025.1654313

**Published:** 2025-07-21

**Authors:** Kamran Sattar, Kate Owen, Bhavani Veasuvalingam

**Affiliations:** ^1^College of Medicine, King Saud University, Riyadh, Saudi Arabia; ^2^University of Warwick Medical School, Coventry, United Kingdom; ^3^IMU University, Kuala Lumpur, Malaysia

**Keywords:** professionalism, wellbeing, curriculum, teaching, cultural differences

Medical professionalism is not governed by static rules; rather, it represents a dynamic and evolving ecosystem. Within the context of our Research Topic, *Nurturing Medical Professionalism in Different Cultural Contexts*, this ecosystem is shaped by the continuous interaction among individual psychology, institutional culture, and societal context.

At the Individual Level (Psychological Foundations), three papers illustrate the internal development of professionalism. Sattar et al. present professionalism as a psychological buffer against burnout, Zeng et al. demonstrate that personality traits influence critical thinking, a core professional skill, and Al-Obiedat et al. extend this understanding to nursing, highlighting empowerment as the psychological fuel for professional satisfaction. Collectively, these studies indicate that professionalism extends beyond behavioral compliance, requiring psychological resilience and empowerment as essential foundations.

The Institutional Level (Hidden and Explicit Curricula) examines how institutions shape professionalism through both formal instruction and the hidden curriculum. Guraya et al.'s PROPER framework directly addresses the hidden curriculum, Sadeq et al.'s systematic review identifies gaps in explicit teaching strategies, and Pandya et al. demonstrate that faculty development can bridge these gaps, rendering professionalism a teachable rather than an assumed construct. This level underscores how institutional culture may either reinforce or erode individual psychological foundations.

The Societal Level (Cultural Context and Justice) expands the analysis to broader cultural and social justice considerations. Iqbal et al. reveal how assessment bias reflects societal inequities, Haque et al. reframe language diversity as an educational opportunity rather than a barrier, and Mohammed et al. illustrate how student-led initiatives can challenge systemic discrimination and promote inclusive professional environments. The following sections provide further elaboration on the specific details derived from these papers.

## Empowerment and quality of work life in nursing

Al-Obiedat et al. examine the psychological empowerment and quality of work life among Jordanian nurses and midwives. Their study reveals a strong positive correlation between empowerment and professional satisfaction, indicating that supportive work environments and autonomy are not only HR concerns but central to professional behavior and job retention. Their findings advocate for investment in nurse empowerment as a strategic approach to enhancing healthcare quality.

## Professionalism, mental health, and coping

Building on this theme of professional wellbeing, Sattar et al. explore how professionalism, mental health, and coping strategies interact among medical students in Malaysia. Their structural equation modeling demonstrates that professionalism buffers against burnout and is positively linked to empathy through adaptive coping strategies. These findings reinforce the idea that professionalism is dynamic, shaped by psychological resilience and institutional support.

## Uncovering the hidden curriculum

Yet professionalism is not shaped solely by formal instruction. Guraya et al. turn attention to the hidden curriculum, unspoken cultural norms that may undermine formal professionalism teaching. Their realist-informed PROPER framework, implemented in two European medical schools, addresses these covert influences. This initiative illustrates that professionalism is best fostered when both the explicit and hidden curricula align to support ethical, reflective practice.

## Assessing professionalism: context matters

In assessing professionalism, context remains crucial. Iqbal et al. evaluate situational judgment tests (SJTs), such as Casper, uncovering that assessor bias and cultural interpretations can influence scoring, even when such tools are intended to measure universal professional attributes. Their work underscores the need for fairness and validity in evaluation, especially across diverse applicant pools.

## What we know about teaching professionalism

A broader view of professionalism instruction is offered by Sadeq et al., who provide a systematic review of educational interventions. While many report positive outcomes, the review highlights methodological inconsistencies and a lack of sustainability in current approaches. These limitations point to the urgent need for contextually responsive, long-term strategies that support meaningful professional identity formation.

## Faculty development: professionalism as a learnable skill

Just as students play a role in shaping professionalism, so too must educators. Pandya et al. detail a faculty development workshop in India that reframed professionalism as a teachable, rather than inherent, quality. Targeted at new faculty, the workshop integrated contemporary theories of professional identity formation. Feedback revealed increased confidence in modeling and teaching professionalism, affirming that faculty development is essential to nurturing these values.

## Personality and critical thinking in China

Further highlighting the complexity of professionalism, Zeng et al. conducted a multicenter study across Chinese medical schools to examine how personality traits and self-differentiation influence critical thinking, a core professional skill. Their findings call for pedagogical strategies tailored to students' psychological profiles and cultural contexts to foster critical thinking effectively.

## Language as an educational resource

Haque et al. contribute another dimension by reframing language barriers as learning opportunities. In a UK-based intervention, non-English consultations in general practice became sites of professional growth. Structured reflection and faculty guidance helped students build empathy and cultural competence, showing how real-world challenges can become powerful educational experiences.

## Challenging islamophobia through student-led teaching innovation

Mohammed et al., in an innovative contribution, present a student-led case-based learning (CBL) initiative that tackled issues of discrimination and microaggressions faced by Muslim medical students in the UK. This intervention, grounded in real-life scenarios, aimed to promote inclusivity and cultural literacy among faculty and students alike. Facilitated by Muslim students with prior experience in curricular innovation, the sessions illuminated challenges such as inadequate prayer spaces, discriminatory clinical attire policies, and weak institutional support systems. Thematic analysis of discussion transcripts, participant feedback, and facilitator reflections revealed five major insights: the need for improved staff and student cultural literacy, more inclusive facilities and policies, and stronger anti-discrimination mechanisms. Significantly, the model's students-as-experts approach helped to rebalance traditional power dynamics, fostering an environment of mutual respect and shared responsibility. This work highlights the role of co-created, culturally sensitive pedagogies in advancing professional identity formation and offers a replicable model for other minority student groups.

## Emerging insights

Professionalism in health professions education is co-constructed emerging through interactions between individuals, institutions, and the sociocultural landscapes they inhabit. It is shaped not only by what is explicitly taught but also by the emotional labor of care, the subtle messages embedded in institutional culture, and the capacity of learners and educators to navigate complexity with integrity. The insights gathered across this Research Topic illuminate how empowerment, psychological resilience, and reflective engagement foster the internalization of professional values. They expose how hidden curricula, linguistic diversity, and culturally laden assessment practices influence the lived experience of becoming a professional. Importantly, these works challenge narrow or prescriptive approaches to teaching professionalism, advocating instead for pedagogies that are attentive to context, sustained over time, and open to student and faculty agency. What emerges is a call for professionalism education that is dialogic, responsive, and anchored in ethical intentionality one that embraces uncertainty, cultivates critical thinking, and reimagines what it means to act professionally in a pluralistic and evolving healthcare world.

## Conclusion

Taken together, these nine contributions challenge us to rethink medical professionalism as a culturally situated, psychologically influenced, and institutionally embedded construct. From empowering nurses to supporting student mental health, from assessing fairly to recognizing hidden curricula, professionalism emerges as a multifaceted endeavor. As editors, educators, and practitioners, we are called not just to define professionalism but to ensure its teaching and assessment are inclusive, sustainable, and context-sensitive. We hope this Research Topic inspires continued innovation and global dialogue in the service of a more human-centered, culturally attuned medical education.

